# Lenvatinib Plus PD-1 Inhibitors as First-Line Treatment in Patients With Unresectable Biliary Tract Cancer: A Single-Arm, Open-Label, Phase II Study

**DOI:** 10.3389/fonc.2021.751391

**Published:** 2021-11-24

**Authors:** Qiyi Zhang, Xingyu Liu, Shumei Wei, Lufei Zhang, Yang Tian, Zhenzhen Gao, Ming Jin, Sheng Yan

**Affiliations:** ^1^ Department of Hepatobiliary and Pancreatic Surgery, The Second Affiliated Hospital Zhejiang University School of Medicine, Hangzhou, China; ^2^ Key Laboratory of Precision Diagnosis and Treatment for Hepatobiliary and Pancreatic Tumor of Zhejiang Province, Hangzhou, China; ^3^ Department of Pathology, The Second Affiliated Hospital Zhejiang University School of Medicine, Hangzhou, China

**Keywords:** biliary tract cancer, lenvatinib, PD-1 inhibitors, first-line treatment, conversion surgery

## Abstract

**Objective:**

We investigated lenvatinib plus programmed cell death-1 (PD-1) inhibitors as a first-line treatment for initially unresectable biliary tract cancer (BTC).

**Methods:**

In this Phase II study, adults with initially unresectable BTC received lenvatinib (body weight ≥60 kg, 12 mg; <60 kg, 8 mg) daily and PD-1 inhibitors (pembrolizumab/tislelizumab/sintilimab/camrelizumab 200 mg or toripalimab 240 mg) every 3 weeks. Primary endpoints were objective response rate (ORR) and safety. Secondary endpoints included surgical conversion rate, disease control rate (DCR), event-free survival (EFS), overall survival (OS) and tumor biomarkers.

**Results:**

Among 38 enrolled patients, the ORR was 42.1% and the DCR was 76.3%. Thirteen (34.2%) patients achieved downstaging and underwent surgery, six of whom (46.2%) achieved a major pathologic response (n=2) or partial pathologic response (n=4) in the primary tumor. In total, 84.2% of patients experienced ≥1 treatment-related adverse event (TRAE), 34.2% experienced a Grade ≥3 TRAE and no treatment-related deaths occurred. After a median follow-up of 13.7 months the median EFS was 8.0 months (95% CI: 4.6–11.4) and the median OS was 17.7 months (95% CI: not estimable).

**Conclusions:**

Lenvatinib plus PD-1 inhibitors showed promising anti-tumor efficacy in patients with initially unresectable BTC and was generally well tolerated.

**Clinical Trial Registration:**

www.chictr.org.cn, ChiCTR2100044476.

## Introduction

Biliary tract carcinomas (BTC) are a group of cancers that include intrahepatic cholangiocarcinoma (ICC), extrahepatic cholangiocarcinoma (ECC) and gallbladder cancer (GBC), and account for 10-15% of primary liver malignancies (1, 2). As a highly aggressive malignant tumor originating from the bile duct epithelium, BTCs are associated with a particularly low life expectancy of around one year. Although radical surgical resection is a potentially curative therapy for BTC, over half of patients have unresectable disease at diagnosis (3). If patients with unresectable BTC are able to achieve adequate downstaging through effective systemic therapy, they may have an opportunity to undergo surgical resection (a ‘conversion resection’) and therefore achieve long-term survival. The conversion therapy treatment strategy is successfully utilized in non-liver cancers and has shown promising results in hepatocellular carcinoma (4). However, despite recent advances in the multidisciplinary treatment of BTC, there remains a lack of effective treatment strategies for achieving secondary resection for patients with this disease.

Gemcitabine combined with cisplatin is currently recommended as the standard first-line therapy for patients with advanced BTC (5). However, the survival outcomes associated with this treatment are suboptimal, with a median overall survival (OS) of approximately 6-8 months (6). In second-line therapy or later, no targeted therapy or immune therapy has yet been approved for advanced BTC. Inhibitors of programmed death-1 (PD-1) and programmed death ligand-1 (PD-L1) have shown promising antitumor efficacy across multiple cancer types (7–9). However, randomized trials of anti-PD-1 and anti-PD-L1 monoclonal antibodies in unresectable or recurrent BTC have so far failed to demonstrate a higher treatment response or survival benefit compared with standard chemotherapies (10). For example, in the KEYNOTE-158 study, patients with advanced BTC receiving pembrolizumab monotherapy achieved an objective response rate (ORR) of 5.8% while the median OS was 7.4 months, and many patients did not achieve any clinical benefit (11, 12). These findings suggest that tumor resistance to anti-PD-1 antibodies limits the proportion of patients with BTC who can benefit from this therapy.

Lenvatinib is a tyrosine kinase (TKI) inhibitor of vascular endothelial growth factor receptor (VEGFR) 1-3, fibroblast growth factor receptor (FGFR) 1-4, platelet derived growth factor receptor a (PDGFRa), RET, and KIT (13). Preclinical studies have demonstrated that lenvatinib can enhance the anti-tumor activity of T lymphocytes in the tumor microenvironment through anti-angiogenic effects, thereby enhancing the anti-tumor effect of anti-PD-1/PD-L1 antibodies (14, 15). Results from mouse models further showed that TKIs combined with PD-1 inhibitors result in greater tumor regression and a higher response rate compared with either treatment alone (16). Clinically, lenvatinib in combination with PD-1 inhibitors has been regarded as a breakthrough therapy in unresectable melanoma (17), hepatocellular carcinoma (18) and renal cell carcinoma (19). Recently, a prospective study (NCT03895970) reported that treatment with lenvatinib plus pembrolizumab (LEP) in patients with refractory BTC resulted in promising antitumor activity (20). However, there are currently no published data on the first-line using combined treatment with TKI inhibitors and PD-1 inhibitors in BTC.

This prospective Phase II trial was conducted to evaluate the efficacy and safety of first-line lenvatinib plus PD-1 inhibitors in patients with initially unresectable BTC and to explore the feasibility of conversion surgery following this therapy.

## Materials and Methods

### Study Design and Patients

This was an open-label, single-center, phase II trial (Chictr.org identifier: ChiCTR2100044476) that included adult (≥18 years) patients with a histologically confirmed diagnosis of biliary tract adenocarcinoma (including ICC, ECC or GBC) that was initially considered unresectable. Initially unresectable BTC was defined as patients for whom R0 resection could not be achieved, even through aggressive surgical procedures, and was determined by a multi-disciplinary team based on imaging evaluation of hepatic artery and portal vein invasion, tumor size, tumor location, remnant liver volume and presence of extrahepatic metastasis. Eligible patients were also required to have ≥1 measurable target lesion according to Response Evaluation Criteria in Solid Tumors (RECIST) v1.1, Child Pugh class A liver function and an Eastern Cooperative Oncology Group (ECOG) performance status of 0 or 1. Patients were excluded if they had received prior chemotherapy, TKI therapy, anti-PD-1 or anti-PD-L1 agents, and had a diagnosis of immunodeficiency or active autoimmune disease or a history of bleeding disorders.

The study protocol was approved by the ethics committee of the Second Affiliated Hospital of Zhejiang University of Medicine and the study was conducted in accordance with the Declaration of Helsinki and principles of Good Clinical Practice. All patients provided written informed consent before inclusion.

### Systemic Therapy

Eligible patients received lenvatinib (body weight ≥60 kg, 12 mg; <60 kg, 8 mg) orally once daily as well as a PD-1 inhibitor intravenously every 3 weeks. Five different PD-1 inhibitors were utilized based on patient preference (pembrolizumab 200 mg, tislelizumab 200 mg, sintilimab 200 mg, camrelizumab 200 mg or toripalimab 240 mg). Treatment was continued until unacceptable toxicity, radiologically confirmed disease progression assessed by RECIST v1.1 or withdrawal of consent.

### Surgical Procedures

For patients who achieved sufficient downstaging during systemic therapy to become eligible for surgery, resection was performed 1 to 3 weeks after the last cycle of treatment. Patients included for conversion resection were evaluated as partial response (PR) or stable disease (SD) for at least 2 months. If patients achieved tumor regression (regressed SD or PR) or lymph node regression, and R0 resection could be achieved with sufficient remnant liver volume but have Grade ≥3 TRAEs, we considered resection for them as soon as possible. However, patients with persistent tumor shrinkage without severe TRAEs will continue to accept the conversion therapy. The criteria for successful surgical conversion included: (1) significant tumor regression (at least partial response or regressed stable disease); (2) Child-Pugh class A liver function; (3) no distant metastasis; (4) R0 resection was possible; (5) ECOG status of 0 or 1; (6) sufficient future liver remnant if hepatic resection was required. Radical resection including systematic lymphadenectomy, partial hepatectomy, combined vascular resection and revascularization were performed according to the extent of tumor invasion during the operation.

### Measurements and Endpoints

Data collected at baseline included patient sex, age, pathological type, clinical TNM staging and carbohydrate antigen 19-9 (CA199) level. The normal value of CA199 was based on our institutional standard. Contrast-enhanced CT or MRI was used to assess the tumor at baseline and every 8 weeks ( ± 2 weeks) thereafter using RECIST v1.1. The primary endpoints were ORR and safety. Secondary outcomes included conversion rate, disease control rate (DCR), event-free survival (EFS), OS and postoperative complications. EFS was defined as the time from initiation of systemic therapy to the occurrence of progressive disease or death from any cause. Safety was assessed throughout the entire study and for 30 days after treatment discontinuation, and during the postoperative period. Treatment-related adverse events (TRAEs) were graded based on the National Cancer Institute Common Terminology Criteria for Adverse Events, version 4.0.

### Pathological Assessments

Surgical tissue specimens were staged according to the American Joint Committee on Cancer (AJCC Cancer Staging Manual. 8th) (21). Hematoxylin and eosin (HE) staining was performed to evaluate the percentage of residual viable tumor in the primary tumor, and ≤10% viable tumor in the treated tumor bed was considered to be a major pathological response (MPR). A partial pathologic response (pPR) was defined as >10% and ≤50% residual viable tumor by chemotherapy criteria while a pathologic nonresponse was defined as >50% residual viable tumor (22).

### Biomarker Analysis

Fine needle aspiration specimens were obtained from each patient before initiation of systemic treatment. Immunohistochemistry was performed to detect the expression of PD-L1 using Dako 22C3 (Dako Monoclonal Mouse Anti-Human PD-L1, Clone 22C3) on tumor biopsy samples. PD-L1 expression was evaluated using isolated tumor cells and certified by a senior pathologist in our hospital. Whole exon sequencing (WES) was conducted using the SureSelect Human All Exon V6 kit (Agilent, Santa Clara, CA, USA). Genomic alterations, including microsatellite stability status, single base substitutions, short and long insertions/deletions (INDELS), copy number variants, and gene rearrangement and fusions, were assessed. Tumor mutation burden (TMB) was determined by analyzing somatic mutations including coding base substitutions and INDELs according to the megabase (Mb).

### Statistical Analysis

Assessment of TRAEs, postoperative complications and feasibility analyses were conducted in all patients who received at least one dose of lenvatinib plus PD-1 inhibitors. Continuous variables were expressed as median (range) and between-group differences were compared using a Student’s t-test or Mann-Whitney U test. Categorical variables were presented as number of patients and associated percentage. The ORR, DCR and duration of response (DoR) and corresponding 95% CIs were calculated using the Clopper-Pearson method. Chi-squared or Fisher exact tests was used to evaluate associations between biomarkers and treatment response. EFS and OS were estimated using the Kaplan-Meier method. All statistical analyses were performed using R software (version 3.6.2) or GraphPad Prism software (version 7).

## Results

### Patients

**Figure 1:**
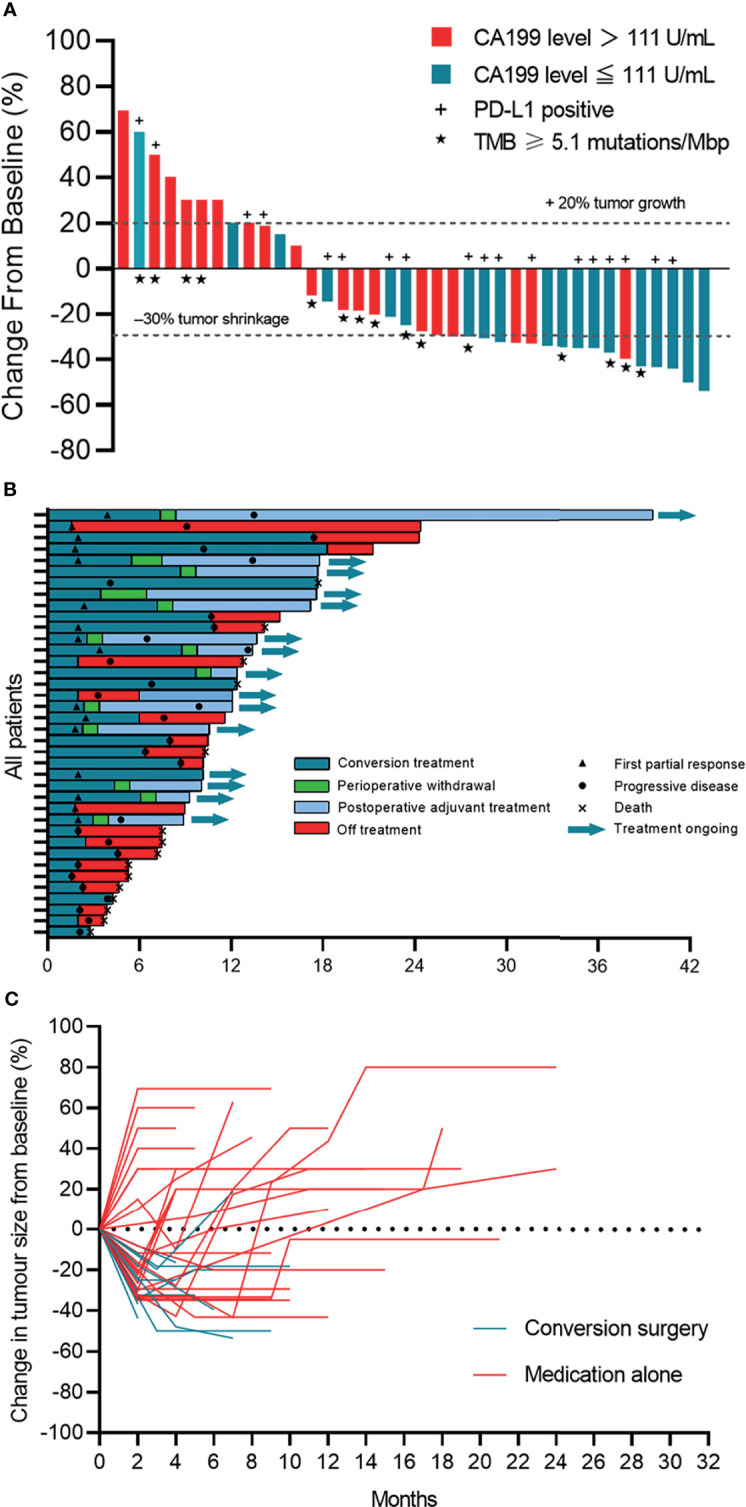
Tumor response. **(A)** Waterfall plot of maximum percent change in tumor size from baseline in each patient as measured by RECIST (version 1.1). **(B)** Time on treatment. **(C)** Longitudinal change in tumor size from baseline. Patients who underwent surgery stopped follow-up after the primary tumor was removed.

Between March 1, 2018 and May 31, 2021, a total of 38 patients were enrolled in the study. Patient demographics and baseline characteristics are summarized in **Table 1**. At the cut-off date for this analysis (May 31, 2021), a total of 23 (60.5%) patients had discontinued treatment and 15 (41.7%) remained on treatment (**Figure S1**). The most common reason for discontinuing both study treatments was confirmed progressive disease or death (n=18). Three patients received second-line combined chemotherapy and two refused to continue treatment due to economic reasons. A total of six patients were still receiving combination treatment with lenvatinib and anti-PD-1 antibodies after surgery despite confirmed tumor relapse. The patient time on treatment is summarized in **Figure 1B**.

**Table 1:** Summary of patient demographics and baseline characteristics.

Characteristic, n (%)	N = 38
Age, years, median (range)	62.50 (57.27-64.52)
Sex	
Male	14 (36.8)
Female	24 (63.2)
PD-1 antibody received	
Pembrolizumab	3 (7.9)
Toripalimab	12 (31.6)
Tislelizumab	11 (28.9)
Sintilimab	11 (28.9)
Camrelizumab	1 (2.6)
ECOG CPS	
0	26 (68.4)
1	12 (31.6)
Tumor subtype	
Intrahepatic cholangiocarcinoma	20 (52.6)
Extrahepatic cholangiocarcinoma	5 (13.2)
Gallbladder cancer	13 (34.2)
TNM stage^a^	
II	5 (13.2)
III	19 (50.0)
IV	14 (36.8)
CA199 level, U/mL	
< 111	18 (50)
≥ 111	18 (50)
Previous therapy	
Radical surgical resection	3 (8.3)
ERCP or PTCD	2 (5.6)

a Clinical staging was based on the 8^th^ edition of the American Joint Committee on Cancer (AJCC) Staging Manual.

CA199, carbohydrate antigen 199; CPS, combined positive score; ECOG, Easter Co-operative Oncology Group; ERCP, endoscopic retrograde cholangiopancreatography; PTCD, percutaneous transhepatic cholangiography and drainage.

### Safety

Dose reductions and treatment discontinuations due to TRAEs were experienced by five (13.9%) and one (2.8%) patients, respectively. Four patients had a lenvatinib dose reduction from 8 mg to 4 mg per day due to lenvatinib-related toxicities. One patient discontinued lenvatinib plus PD-1 inhibitor because of treatment-related cerebral hemorrhage.

During the study, 84.2% of patients experienced ≥1 TRAE, and there were no treatment-related deaths (**Table S1**). Most TRAEs were of a low grade and were easily managed. The most common TRAEs of any Grade were fatigue (n=14), anorexia (n=8), alanine aminotransferase (ALT) elevation (n=7) or aspartate aminotransferase (AST) elevation (n=7), rash (n=6), hypertension (n=5) and hoarseness (n=5). Grade ≥3 TRAEs occurred in 34.2% of patients and the most common were fatigue (n=5) and hypertension (n=3). One patient experienced Grade 4 cerebral hemorrhage caused by hypertension, which was the most serious TRAE observed, although the patient was successfully treated for this adverse event.

### Radiographic Response Evaluation

All patients had at least one radiological evaluation. A total of 16 (42.1%, 95% CI: 25.7% to 58.6%) patients achieved a partial response, 13 (34.2%, 95% CI: 18.4% to 50.0%) achieved stable disease and no patients achieved a complete response (**Table 2** and **Figure 1A**). The ORR was 42.1% (95% CI: 25.7% to 58.6%). Among the 16 patients who achieved a partial response, nine (56.3%) were confirmed as an objective response. The DCR was 76.3 (95% CI: 62.2% to 90.5%).

**Table 2:** Summary of tumor response and survival outcomes.

Therapeutic response assessment	N = 38
ORR^a^, % (95% CI)	42.1 (25.7 to 58.6)
Confirmed ORR^a,b^, % (95% CI)	9 (23.7, 9.5 to 37.8)
Best overall response^a,b^, n (%) [95% CI]	
CR	0
PR	16 (42.1) [25.7 to 58.6]
SD	13 (34.2) [18.4 to 50.0]
PD	9 (23.7) [9.5 to 37.8]
Conversion rate, n (%) [95% CI]	13 (34.2) [18.4 to 50.0]
Conversion time, months, median (95% CI)	5.5 (3.8 to 7.1)
DCR^b^, % (95% CI)	76.3 (62.2 to 90.5)
EFS^c^, months, median (95% CI)	8.0 (4.6 to 11.4)
6-month EFS rate, % (95% CI)	63.2 (47.1 to 79.2)
1-year EFS rate, % (95% CI)	21.1 (7.5 to 34.6)
EFS for patients who underwent surgery, months, median (95% CI)	13.5 (13.0 to 14.0)
EFS for patients who did not undergo surgery, months, median (95% CI)	4.6 (0.8 to 8.4)
OS^c^, months, median (95% CI)	17.7 (NR)
6-months OS rate, % (95% CI)	81.6 (68.7 to 94.5)
1-year OS rate, % (95% CI)	47.4 (30.7 to 64.0)
OS for patients who underwent surgery, months, median (95% CI)	NR
OS for patients who did not undergo surgery, months, median (95% CI)	12.4 (8.5 to 16.3)

a Treatment response was evaluated according to RECIST v1.1.

b Calculated using exact method of binomial distribution (Clopper-Pearson method).

c Kaplan-Meier method was used for estimating EFS and OS.

CI, confidence interval; CR, complete response; DCR, disease control rate; EFS, event-free survival; ORR, objective response rate; PD, progressive disease; PR, partial response; NR, not reached; OS, overall survival; SD, stable disease.

### Surgery

**Figure 2:**
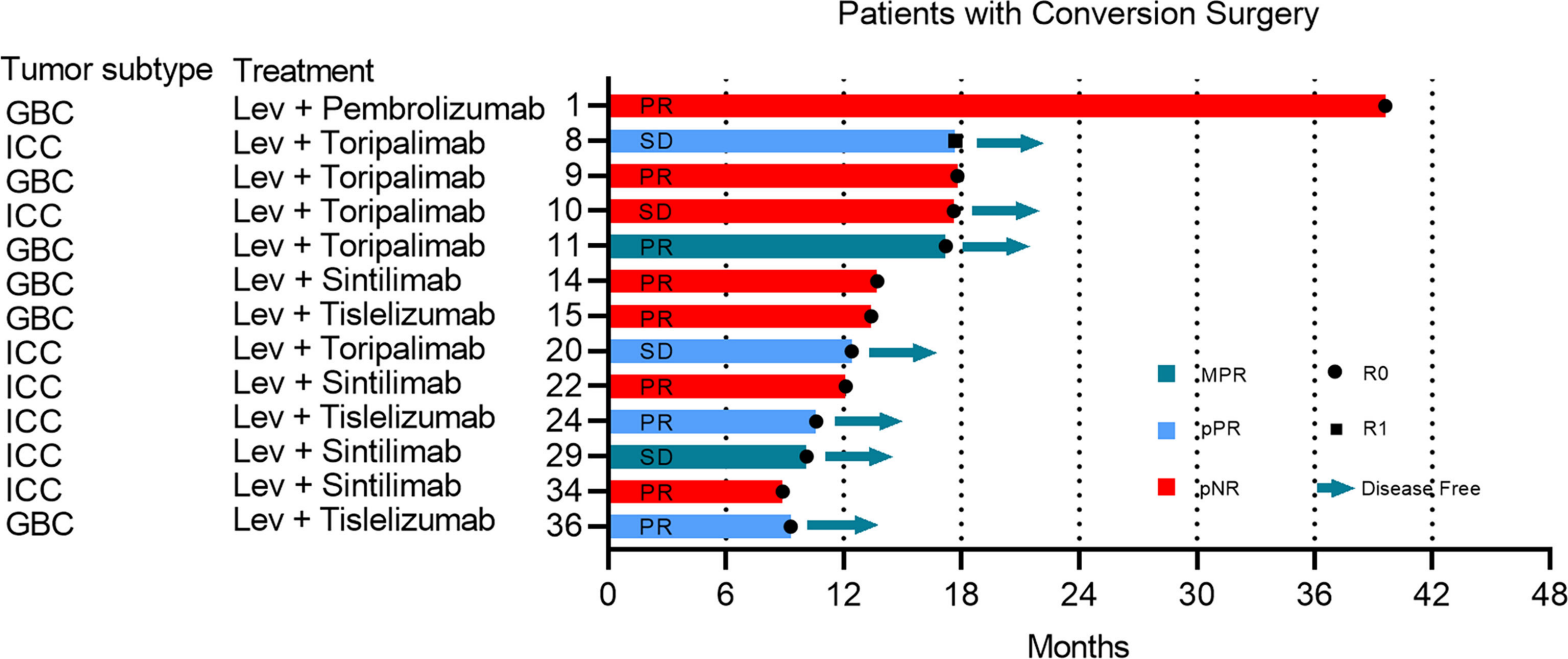
The complete course of conversion treatment and post-surgery outcome. No more than 10% of viable tumor in the treated tumor beds were considered to have had a major pathological response (MPR). Partial pathologic response (pPR) was defined as more than 10% and less than 50% residual viable tumor by chemotherapy criteria while pathologic nonresponse (pNR) was defined as more than 50% residual viable tumor. The preoperative radiographic response of each patient was marked in the bar. ICC, intrahepatic cholangiocarcinoma; GBC, gallbladder cancer; PD, progressive disease; PR, partial response; SD, stable disease; Lev, lenvatinib.

**Figure 3:**
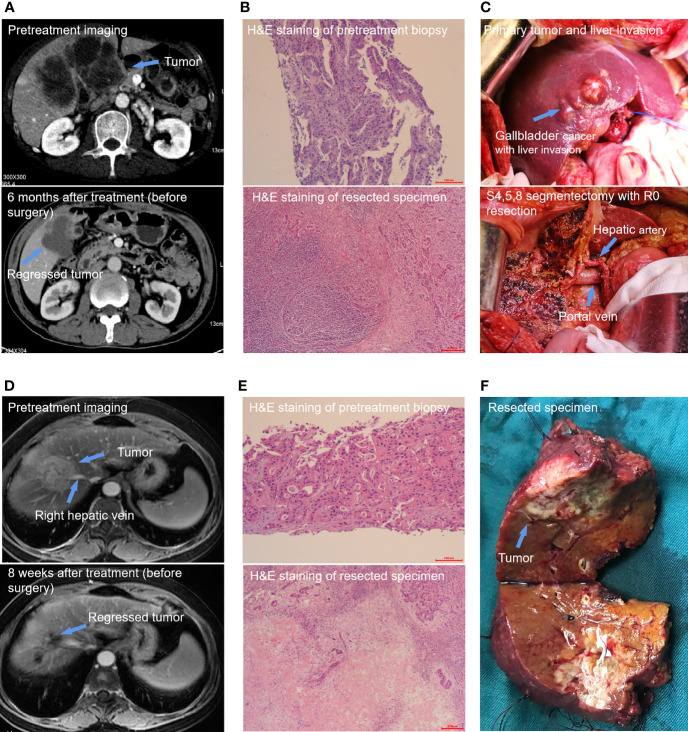
Two special cases report. (1) Patient 11 was a 65-year-old male patient with stage IIIB gallbladder cancer. Pretreatment contrast-enhanced computed tomography (CT) imaging of the abdomen showed a huge tumor including the primary tumor and liver invasion. The tumor was significantly shrunk after 6 months of conversion treatment **(A)**. Hematoxylin and eosin staining of resected specimen showed a MPR and a plenty of lymphocyte infiltration **(B)**. S4,5,8 segmentectomy with R0 resection **(C)**. (2) Patient 24 was a 65-year-old female patient with stage II intrahepatic cholangiocarcinoma. The pretreatment Magnetic Resonance Imaging (MRI) imaging showed a primary tumor mass of 5.2 cm in diameter and has invaded the main branch of the right hepatic vein. A scan performed before surgery showed that most of the primary tumors had appeared necrosis and shrunk significantly **(D)**. The pathologic images shown are representative sections of the patient before conversion therapy and large amount of post-treatment necrosis and tumor-infiltrating lymphocytes and macrophages were found in the primary tumor postoperation **(E)**. Specimen with R0 resection **(F)**.

Of 38 evaluable patients, 34.2% (n=13) achieved adequate tumor volume reduction were considered eligible for resection (**Figure S2**). The median conversion time from initiation of systemic therapy to surgery was 5.5 months (95% CI: 3.8 to 7.1). Among patients who underwent conversion surgery, 12 (92.3%) achieved a R0 resection, and one underwent palliative resection due to abdominal tumor distant metastasis (**Figure 2**). Representative images from two patients who successfully underwent conversion surgery and achieved good postoperative outcomes are presented in **Figure 3**.

Of the patients who underwent resection, six experienced postoperative complications, including two cases of biliary leakage, two cases of pleural effusion, one case of delayed liver function recovery and one case of upper gastrointestinal bleeding. All patients undergoing surgery were successfully discharged after postoperative care.

### Follow-up

**Figure 4:**
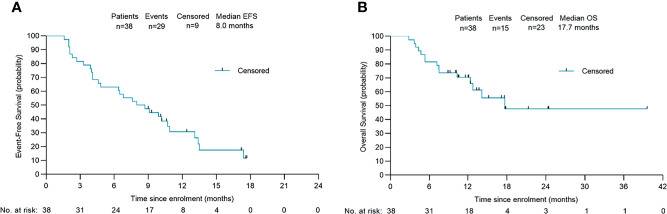
Survival outcomes of 38 patients. Kaplan-Meier plots of overall event-free survival **(A)** and overall survival **(B)**. Probability of survival is shown at indicated time points. Numbers of patients at risk at indicated time points are shown below the x-axis. Censored patients are marked with a vertical line in the graph. EFS, event-free survival; OS, overall survival.

After a median follow-up of 13.7 (95% CI: 9.7 to 17.8) months, the 1-year OS rate was 47.4% (18/38), and 65.8% of patients were still alive. The median EFS was 8.0 months (95% CI: 4.6 to 11.4) and the median OS was 17.7 months (95% CI: not estimable) (**Figure 4**). Among the 13 patients who underwent conversion surgery, the median EFS was 13.5 months (95% CI: 13.0 to 14.0).The median recurrence-free survival (RFS) and median OS were not reached (**Table S2**). Among patients who only received systemic therapy, the median EFS was 4.6 months (95% CI: 0.8 to 8.4) and the median OS was 12.4 months (95% CI: 8.5 to 16.3). Compared to patients receiving only systemic therapy, patients who successfully achieved conversion resection had a longer EFS and OS (**Table 2**). One patient who underwent conversion surgery had survived for 39.0 months as of the cut-off. The change in tumor size for patients in the conversion surgery group and no-surgery group is shown in **Figure 1C**.

### Pathologic Findings

Among the 13 patients who underwent a conversion resection, two (15.4%) achieved a MPR in the primary tumor, and four (30.8%) achieved a pPR (**Figure 2**). No patient achieved a complete pathological response (no viable tumor cells). The median degree of pathological regression in the primary tumor were -30% (range: -26.8% to -65.6%). No post-surgical relapse was observed in patients who achieved a MPR or pPR. However, five (71.4%) patients who were pathological non-responders experienced disease recurrence. Pathological analysis of resected tumor specimens revealed varying degrees of post-treatment necrosis and treatment-related immune activation. In primary tumors categorized as MPR or pPR, we observed a large number of tumor-infiltrating lymphocytes and macrophages, which were especially notable in the surrounding adjacent tissue. However, these tumor immune response-related cells were rarely observed in patients who were pathologic non-responders. Necrosis was found mainly in the middle area of the tumor and finally replaced by fibrosis (**Figures 3B, E**).

### PD-L1 Expression Analysis

**Figure 5:**
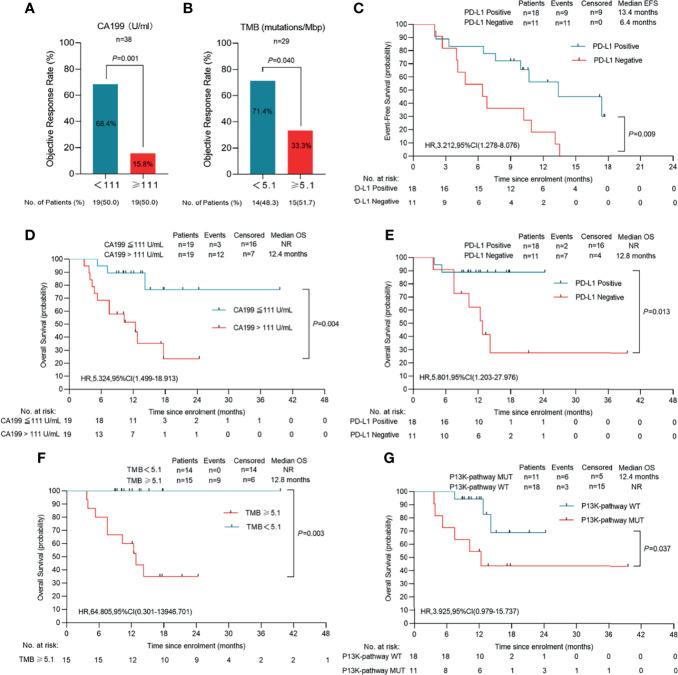
Clinical response in relation to tumor biomarkers in patients with initial unresectable BTC. **(A)** The cutoff value of CA199(CA199 = 111 U/mL) is three times the normal value of our institution (n=38). **(B)** TMB was calculated by summing up somatic mutations within the coding regions by whole-exon sequencing. A TMB of 5.1 mutations per million base pairs (Mbp) was the cutoff value (n=29). **(C)** Event-free survival of patients of PD-L1 positive or PD-L1 negative (n=29). **(D)** Overall survival of patients of CA199 ≤ 111 U/ml and CA199 > 111U/ml (n=38). **(E)** Overall survival of patients of PD-L1 positive or PD-L1 negative (n=29). **(F)** Overall survival of patients of TMB<5.1 mutations/Mbp or TMB ≥ 5.1 mutations/Mbp (n=29). **(G)** Overall survival of patients of P13K-pathway wide type and P13K-pathway mutation (n=29). Probability of survival is shown at indicated time points. Censored patients are marked with a vertical line in the graph. Numbers of patients at risk at indicated time points are shown below the x-axis. NR, not reached.

PD-L1 expression was evaluated in pretreatment biopsy samples obtained from 29 patients. Immunohistochemistry showed that objective responses were achieved by 10 (61.1%) of 18 patients with positive PD-L1 (combined positive score [CPS] ≥1%) and five (45.5%) of 11 patients with negative PD-L1 (CPS <1%) (**Figure S3A**). Consequently, patients with positive PD-L1 expression showed significantly prolonged survival outcomes for both event free survival (EFS) (*P*=0.009) and OS (*P*=0.013), suggesting that PD-L1 expression is a potential prognostic factor (**Figures 5C, E**). Moreover, in the subgroup of patients who underwent resection, four PD-L1 positive samples were identified in the five patients (80.0%) who achieved a MPR or pPR, while only 57.1% of patients with pathologic non-response were positive for PD-L1. In an immune response analysis, patients with positive PD-L1 expression were shown to have more tumor infiltrating lymphocytes clustered around the tumor (**Figure 3B**).

### Genomic Analysis

**Figure 6:**
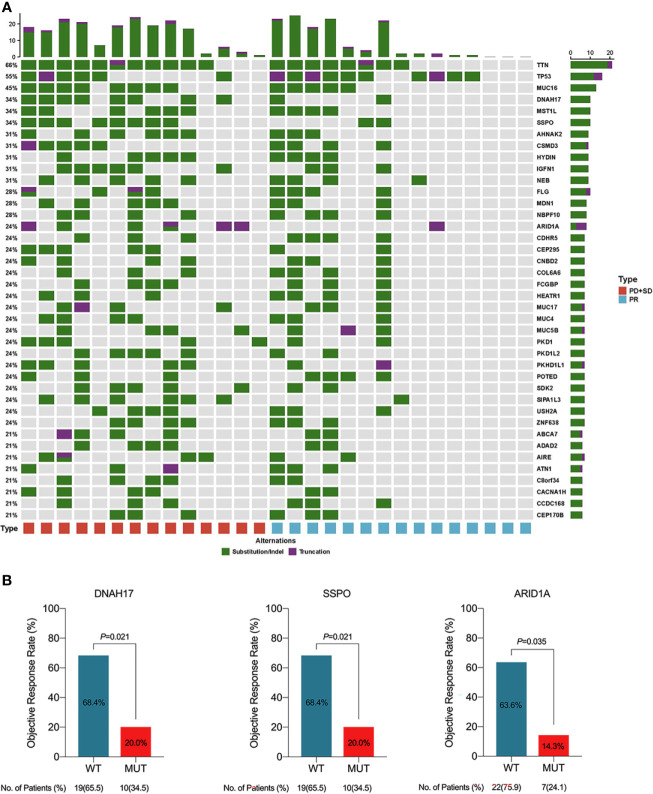
**(A)** Genetic alternations and frequencies identified by whole exome sequencing (WES) from 29 available patients. **(B)** Clinical response in relation to gene mutations in patients with initial unresectable BTC. PD, progressive disease; PR, partial response; SD, stable disease; WT, wide type; MUT, mutation.

We performed WES on pre-treatment tumor samples obtained from 29 patients who had adequate available tissue. The relationship between clinical response to lenvatinib plus anti-PD-1 therapy and underlying molecular profiles was investigated. A total of 3124 mutations were detected. Statistical analysis showed that mutations in DNAH17, SSPO or ARID1A were significantly associated with low ORR (DNAH17, 6.9% vs 44.83%, *P*=0.02; SSPO, 6.90% vs 44.83%, *P*=0.02; ARID1A, 3.45% vs 48.28%, *P*=0.04) (**Figure 6B**). Prognosis analysis revealed that patients with PI3K-pathway mutations had shorter EFS (median EFS, 6.5 months vs 10.9 months, *P*=0.074) (**Figure S4C**) and OS (median OS, 12.4 months vs not reached, *P*=0.037) (**Figure 5G**) compared with the PI3K-pathway wild-type group. Genetic alterations and frequencies identified by WES are summarized in **Figure 6A** and **Table S3**.

### TMB

TMB was determined by analyzing somatic mutations within the coding region of the human genome though WES. The median TMB was 5.10 muts/Mb in 29 patients with available data. Ten (71.4%) of 14 patients with a low TMB (median 5.10 as the cutoff value) and five (33.3%) of 15 patients with a high TMB achieved objective responses (**Figure 5B**). In addition, patients with a low TMB had a significantly longer OS than those with a high TMB (*P=*0.003) (**Figure 5F**).

### Other Biomarker Analyses

Chi-squared showed that pretreatment serum CA199 level was associated with treatment response. Using 111 U/mL as the cutoff value, patients with lower CA199 levels had a significantly higher ORR (68.4%) (**Figure 5A**) and prolonged OS compared to the high CA199 level group (*P*=0.028) (**Figure 5D**). In the low CA199 level group, 10 of 18 patients (55.6%) underwent resection, and no deaths had occurred in these patients at the data cut-off.

Interestingly, compared with patients with ECC or ICC, those with GBC had a higher ORR (61.5%) and a higher surgical conversion rate (46.2%). However, given the small sample size, further study is warranted in a larger cohort of GBC.

## Discussion

Locally advanced and metastatic BTCs are generally considered unresectable and lack effective treatment options. Despite the progress made in other cancers, no TKI or PD-1 inhibitor has been approved for the treatment of advanced BTC to date. Preclinical evidence suggests that combined treatment with a TKI inhibitor and anti-PD-1 or anti-PD-L1 antibodies induces additive antitumor effects. Furthermore, a clinical study in refractory BTC also reported higher ORR and longer OS with TKI or PD-1 inhibitor combination therapy compared with prior findings for TKI or PD-1 inhibitor monotherapy (20). The present study provides clinical evidence that combination therapy with a TKI plus PD-1 inhibitor in the first-line treatment of advanced BTC may provide a robust anti-tumor effect and allow a proportion of patients to achieve downstaging and conversion to surgical treatment.

To our knowledge, this is the first trial of lenvatinib plus PD-1 inhibitors for the first-line treatment of BTC. We found that this combination therapy is relatively well tolerated, with TRAEs experienced by 84.2% of patients and no treatment-related deaths. In addition, a total of 34.2% of patients experienced Grade ≥3 TRAEs, which was lower than reported in a previous study of LEP in 32 Chinese patients with refractory BTC in which 100% of patients experienced TRAEs and 59.3% experienced Grade ≥3 AEs (20). The most common TRAEs reported with LEP in this previous study were fatigue, hypertension and anorexia, which were similar with those observed in the present study. Furthermore, of the 13 patients in the present study who underwent surgery, only six experienced postoperative complications, while all patients who underwent surgery were successfully discharged after postoperative care. Generally, the combined regimen of lenvatinib plus PD-1 inhibitors was well tolerated and all toxicities were manageable.

In terms of efficacy, we found a DCR of 76.3% and an ORR of 42.1%, with a median EFS of 8.0 months, a median OS of 17.7 months and a 1-year OS rate of 47.4%. These findings suggest that combination therapy exerts a better anti-tumor effect than TKI or PD-1 inhibitor monotherapy in patients with advanced BTC. Furthermore, the surgical conversion rate of this cohort reached 34.2% and the successful R0 resection rate was 31.6%. There have been no prior reports of conversion therapy with TKIs or PD-1 inhibitors in BTC; however, previous studies demonstrated that chemotherapy can lead to downstaging and conversion to surgery. A recent systematic review including patients from 10 trials indicated a surgical conversion rate of 17.3% (27/132), and that 23 of the 27 patients who underwent surgery were alive at the last reported follow-up of this study (23). Notably, the conversion rate reported for chemotherapy in this prior systematic review was lower than observed in our study (17.3% vs 31.6%). A further study conducted in South Korea assessed chemoradiotherapy (CRT) for downstaging unresectable ICC to resectable lesions and reported an ORR of 25% and conversion rate of 12.5% (24). Eight patients in this study were able to receive a curative resection after CRT and showed significantly improved OS compared to patients treated with CRT alone (3-year OS: 50% vs. 11.2%, respectively, *P*=0.012). Although patients in this prior study who were able to undergo surgery achieved prolonged survival, the conversion rate was relatively low compared with the present study in which the R0 resection rate was 31.6%. Therefore, the combination of lenvatinib plus PD-1 inhibitors outperformed both chemotherapy and CRT in terms of conversion to surgical resection for patients with advanced BTC.

Basic laboratory research has revealed that the immunologic effects of the PD-1 pathway on T cell priming, effector function and exhaustion suggest distinct mechanisms underlying surgical conversion with immunotherapy versus chemotherapy. Chemotherapy achieves downstaging and conversion by reducing tumor burden preoperatively, whereas immunotherapy can enhance systemic immunity against tumor antigens, thereby also inhibiting postoperative recurrence by eliminating micrometastatic tumors (25). Moreover, the inhibition of tyrosine kinases can enhance the function of T lymphocytes in the tumor microenvironment through anti-angiogenic effects, thereby enhancing the anti-tumor effect of anti-PD-1/PD-L1 antibodies (26). Therefore, combining TKIs with PD-1 inhibitors has been shown to promote the anti-tumor effect of T cells in the immune system, whereas chemotherapy inhibits this mechanism by depleting regulatory T cells. This may explain why the clinical efficacy of lenvatinib plus PD-1 inhibitors in this study exceeded that reported in studies of conversion therapy in BTC using chemotherapy.

We evaluated the pathological response of 13 surgically resected tumor specimens. Although the MPR rate was low (15.4%) on histological examination, there was an association with prognosis. Among patients with primary tumor shrinkage of more than 50% (MPR and pPR), no postsurgical relapse was observed by the cut-off date for this analysis, while 71.4% (5/7) of those with a pathologic non-response experienced tumor recurrence. One patient who experienced stable disease assessed by RECIST 1.1 achieved 95% tumor shrinkage in postoperative pathological analysis. A correlation between pathologic response and improved recurrence-free survival or OS has been shown in neoadjuvant studies for several cancer types (27–29). These findings prove that postoperative pathological analysis has certain advantages in predicting postoperative tumor recurrence, and this has been a longstanding surrogate endpoint in studies of advanced BTC.

The identification of biomarkers to evaluate tumor response in the conversion setting represents an important secondary aim of this study. In this regard, we found that histopathological type of BTC was associated with treatment response and prognosis. Patients with GBC (n=13) had an ORR of 61.5% which was higher than patients with ICC (40%) or ECC (0%). Six patients (46.2%) with GBC underwent resection, while none with ECC achieved an objective response or converted to surgical resection. Although this result is interesting, considering the small sample size and possible selection bias, further study in a larger cohort of BTC is needed.

CA199 is a commonly used biomarker for predicting recurrence of BTC after neoadjuvant chemotherapy. Lehrke et al. retrospectively analyzed data from 132 patients with perihilar cholangiocarcinoma who underwent liver transplantation after neoadjuvant chemoradiotherapy. They found that the postoperative recurrence and mortality rates of patients with CA199 level ≥200 U/L were 2.3 times and 2.4 times that of patients with CA199 level <200 U/L, respectively (30). Similarly, in the present study, we found that pretreatment CA199 level was closely related to response to therapy. Patients with low CA199 levels had a higher ORR (68.4%) and OS than the high CA199 group. These findings suggest that CA199 level could be used to screen patients with BTC and identify those who are likely to respond to combination therapy.

PD-L1 expression has been associated with the response to immunotherapy in various cancers. Lin et al. reported a trial of LEP in patients with refractory BTC and found that positive PD-L1 expression in tumors pre-treatment was significantly associated with a higher clinical benefit rate and improved PFS and OS (20). In the present study, the subgroup of patients with positive PD-L1 expression achieved a higher ORR and had a significantly prolonged EFS and OS compared with patients who had negative PD-L1 expression, which was consistent with the study reported by Lin et al. Furthermore, patients with positive PD-L1 expression who underwent conversion resection had more tumor infiltrating lymphocytes clustered around the tumor. These results suggest that PD-L1 expression is a potential prognostic factor for the treatment of BTC with combined lenvatinib plus PD-1 inhibitors.

We evaluated the predictive value of TMB for response to combination therapy. Unexpectedly, using the median TMB as the cutoff value, patients with lower TMB exhibited a better objective response and longer OS compared with patients with higher TMB. Although these findings are contrary to many previous reports, similar trends have been found in some recent studies. For example, Wang et al. reported a phase II clinical trial of toripalimab in recurrent or metastatic nasopharyngeal carcinoma. They found that none of the patients with TMB value over 10 muts/Mb achieved an objective response to toripalimab and also had a short PFS (1.68-3.25 months) and OS (2.30-9.56 months) (31). Although our study found no statistically significant differences between the low- and high-TMB groups, there was a trend towards longer OS for patients with lower TMB and this is supported by previous research. However, it should be noted that these results may be due to selection bias. More research is required to confirm the objectivity of these results.

We attempted to identify genomic biomarkers for response to lenvatinib plus PD-1 inhibitors in patients with advanced BTC. WES of 29 patients demonstrated that DNAH17, SSPO or ARID1A alterations were significantly associated with poor response. Although there have been prior reports of abnormal expression of DNAH17 and SSPO genes in tumors, there is a lack of systematic research of the association between these abnormalities and outcomes of anti-tumor therapy. Despite this, the mutation of ARID1A in the present study was consistent with previous reports. Hu et al. found that loss of ARID1A activated Ang2-dependent angiogenesis and promoted hepatocellular carcinoma progression. In addition, ARID1A alterations are known to confer sensitivity to anti-angiogenic therapy (32). As a tumor suppressor gene, the impact of genomic amplification in ARID1A on anti-angiogenic function and immunotherapy requires further investigation in BTCs. We also identified P13K pathway mutations as another potential biomarker of prognosis. The association between hyperactivity and activation of the P13K pathway and response to radiotherapy and chemotherapy has been previously reported and is a known negative prognostic factor for various cancer types (33). Consistent with these previous findings, in our study, patients with mutations in the P13K pathway had worse outcomes than the wild type group. However, due to the heterogeneity of tumors, more studies in BTC are required to validate this result.

The key limitations of this trial include the following. (1) Five different PD-1 inhibitors were used and differences in drug mechanisms cannot be ignored. However, we found no significant differences in treatment efficiency between the different treatment regimens. (2) This trial had a non-randomized design with a relatively small number of patients enrolled, which may have led to participant bias and selection bias. Large-scale studies with long-term follow-up are needed to verify the effects of conversion therapy and discover the best biomarkers for predicting response. (3) Not all patients had enough pre-treatment biopsy tissue for PD-L1 and whole-genome sequencing, which limited the accuracy of tumor marker exploration.

## Data Availability Statement

The datasets presented in this study can be found in online repositories. The names of the repository/repositories and accession number(s) can be found in the article/**Supplementary Material**.

## Ethics Statement

The studies involving human participants were reviewed and approved by the ethics committee of the Second Affiliated Hospital of Zhejiang University of Medicine. The patients/participants provided their written informed consent to participate in this study.

## Authors Contributions

QZ and XL wrote the manuscript. LZ, YT, ZG, and MJ helped to collect patient data. SW provided the pathologic analysis. SY designed the research and supervised the report. All authors contributed to the article and approved the submitted version.

## Funding

This work was supported by the Medical Science and Technology Foundation of Zhejiang Province, No.2020KY571; the “Zhejiang Provincial Program for the Cultivation of High-level Innovative Health talents”; the National Natural Science Foundation of China, No.81902405 and No.82000619.

## Conflict of Interest

The authors declare that the research was conducted in the absence of any commercial or financial relationships that could be construed as a potential conflict of interest.

## Publisher’s Note

All claims expressed in this article are solely those of the authors and do not necessarily represent those of their affiliated organizations, or those of the publisher, the editors and the reviewers. Any product that may be evaluated in this article, or claim that may be made by its manufacturer, is not guaranteed or endorsed by the publisher.
