# Supplementary material for: Lenvatinib Plus PD-1 Inhibitors as First-Line Treatment in Patients With Unresectable Biliary Tract Cancer: A Single-Arm, Open-Label, Phase II Study
Source: Front Oncol. 2021 Nov 24;11:751391. doi: 10.3389/fonc.2021.751391 (PMC8651538; doi:10.3389/fonc.2021.751391)
Supplement: Supplementary file 1 [file DataSheet_1.docx]

**Lenvatinib Plus PD-1 Inhibitors as First-line Treatment in Patients with Unresectable Biliary Tract Cancer: A Single-arm, Open-Label, Phase II Study**

Qiyi Zhang, Xingyu Liu, Shumei Wei, Lufei Zhang, Yang Tian, Zhenzhen Gao, Ming Jin, Sheng Yan

**Table of contents**

Table S1……………………………………………………………………….. 2

Table S2……………………………………………………………………….. 3

Table S3……………………………………………………………………….. 4

Fig. S1………………………………………………………………………….5

Fig. S2………………………………………………………………………….6

Fig. S3………………………………………………………………………….7

Fig. S4………………………………………………………………………….8

Table S1. Safety summary

| Treatment-Related Adverse Events, n (%) | All Grades^a^ | Grade 1 | Grade 2 | Grade 3 | Grade 4 |
| --- | --- | --- | --- | --- | --- |
| All | 32 (84.2) | 28 (73.7) | 19(50.0) | 12 (31.6) | 1 (2.6) |
| Fatigue | 14 (36.8) | 7 (18.4) | 2 (5.3) | 5 (13.2) | -- |
| Anorexia | 8 (21.1) | 8 (21.1) | -- | -- | -- |
| ALT elevation | 7 (18.4) | 7 (18.4) | -- | -- | -- |
| AST elevation | 7 (18.4) | 6 (15.8) | 1 (2.6) | -- | -- |
| Rash | 6 (15.8) | -- | 4 (10.5) | 2 (5.3) | -- |
| Hypertension | 5 (13.2) | 1 (2.6) | -- | 3 (7.9) | 1 (2.6) |
| Hoarseness | 5 (13.2) | 5 (13.2) | -- | -- | -- |
| Leukopenia | 4 (10.5) | 2 (5.3) | 2 (5.3) | -- | -- |
| Erythrocytopenia | 4 (10.5) | 4 (10.5) | -- | -- | -- |
| Muscle soreness | 4 (10.5) | 1 (2.6) | 3 (7.9) | -- | -- |
| Pruritus | 4 (10.5) | -- | 2 (5.3) | 2 (5.3) | -- |
| Hand and foot syndrome | 4 (10.5) | 1 (2.6) | 3 (7.9) | 1 (2.6) | -- |
| Anemia | 3 (7.9) | 3 (7.9) | -- | -- | -- |
| Nausea | 3 (7.9) | -- | 3 (7.9) | -- | -- |
| Fever | 3 (7.9) | 1 (2.6) | 2 (5.3) | -- | -- |
| Diarrhea | 3 (7.9) | 2 (5.3) | 1 (2.6) | -- | -- |
| Hypothyroidism | 3 (7.9) | -- | 3 (7.9) | -- | -- |
| Alkaline phosphatase increased | 3 (7.9) | 3 (7.9) | -- | -- | -- |
| Weight loss | 3 (7.9) | 3 (7.9) | -- | -- | -- |
| Alopecia | 3 (7.9) | 3 (7.9) | -- | -- | -- |

^a^ Treatment-related adverse events were graded according to the National Cancer Institute Common Terminology Criteria for Adverse Events, version 4.0.

ALT, alanine aminotransferase; AST, aspartate aminotransferase.

**Table S2.** Summary of patient demographics and baseline characteristics

| Case | Gender | Age (years) | PD-1 antibody | Pathology type | Pre-treatment stage^a^ | Treatment response | PD-L1 expression | Conversion time(months) | Post-surgery recurrence(months) | RFS(months) | OS(months) |
| --- | --- | --- | --- | --- | --- | --- | --- | --- | --- | --- | --- |
| 1 | Female | 37 | Pembrolizumab | GBC | IIIB | PR | Negative | **7.4** | Yes | 6.1 | 39.0 |
| 8 | Male | 50 | Toripalimab | ICC | IV | SD | Positive | **8.7** | No | 9.0 | 17.7 |
| 9 | Female | 51 | Toripalimab | GBC | IIIB | PR | Positive | **5.5** | Yes | 7.9 | 17.8 |
| 10 | Female | 71 | Toripalimab | ICC | II | SD | Positive | **3.5** | No | 14.1 | 17.6 |
| 11 | Male | 65 | Toripalimab | GBC | IIIB | PR | Positive | **7.2** | No | 10.0 | 17.2 |
| 14 | Female | 60 | Sintilimab | GBC | IV | PR | Positive | **2.6** | Yes | 3.9 | 13.7 |
| 15 | Male | 63 | Tislelizumab | GBC | IIIA | PR | Negative | **8.8** | Yes | 4.2 | 13.4 |
| 20 | Female | 61 | Toripalimab | ICC | IIIB | SD | Positive | **9.7** | No | 2.7 | 12.4 |
| 22 | Male | 57 | Sintilimab | ICC | IIIB | PR | Positive | **2.4** | Yes | 7.5 | 12.1 |
| 24 | Female | 65 | Tislelizumab | ICC | II | PR | Positive | **2.3** | No | 8.3 | 10.6 |
| 29 | Female | 44 | Sintilimab | ICC | IIIB | SD | Positive | **4.4** | No | 5.7 | 10.1 |
| 34 | Male | 65 | Sintilimab | ICC | Ⅱ | PR | Negative | **6.1** | Yes | 2.6 | 8.9 |
| 36 | Female | 47 | Tislelizumab | GBC | IV | PR | Positive | **2.2** | No | 3.1 | 9.3 |

^a^Clinical staging was based on the 8^th^ edition of the American Joint Committee on Cancer (AJCC) Staging Manual.

ICC, intrahepatic cholangiocarcinoma; GBC, gallbladder cancer; PR, partial response; SD, stable disease; RFS, recurrence-free survival; OS, overall survival.

Table S3. The relationship between gene expression and treatment efficacy (n = 29)

| **Variables** | **Total (n=29)** | **PD+SD (n=14)** | **PR (n=15)** | **P-value** |
| --- | --- | --- | --- | --- |
| TTN, n (%) |  |  |  | 0.2451 |
| MUT | 19 (65.52) | 11 (78.57) | 8 (53.33) |  |
| WT | 10 (34.48) | 3 (21.43) | 7 (46.67) |  |
| TP53, n (%) |  |  |  | 0.3603 |
| MUT | 16 (55.17) | 6 (42.86) | 10 (66.67) |  |
| WT | 13 (44.83) | 8 (57.14) | 5 (33.33) |  |
| MUC16, n (%) |  |  |  | 0.3603 |
| MUT | 13 (44.83) | 8 (57.14) | 5 (33.33) |  |
| WT | 16 (55.17) | 6 (42.86) | 10 (66.67) |  |
| DNAH17, n (%) |  |  |  | 0.0209 |
| MUT | 10 (34.48) | 8 (57.14) | 2 (13.33) |  |
| WT | 19 (65.52) | 6 (42.86) | 13 (86.67) |  |
| MST1L, n (%) |  |  |  | 0.4497 |
| MUT | 10 (34.48) | 6 (42.86) | 4 (26.67) |  |
| WT | 19 (65.52) | 8 (57.14) | 11 (73.33) |  |
| SSPO, n (%) |  |  |  | 0.0209 |
| MUT | 10 (34.48) | 8 (57.14) | 2 (13.33) |  |
| WT | 19 (65.52) | 6 (42.86) | 13 (86.67) |  |
| AHNAK2, n (%) |  |  |  | 0.2451 |
| MUT | 9 (31.03) | 6 (42.86) | 3 (20.00) |  |
| WT | 20 (68.97) | 8 (57.14) | 12 (80.00) |  |
| CSMD3, n (%) |  |  |  | 0.6999 |
| MUT | 9 (31.03) | 5 (35.71) | 4 (26.67) |  |
| WT | 20 (68.97) | 9 (64.29) | 11 (73.33) |  |
| HYDIN, n (%) |  |  |  | 0.6999 |
| MUT | 9 (31.03) | 5 (35.71) | 4 (26.67) |  |
| WT | 20 (68.97) | 9 (64.29) | 11 (73.33) |  |
| IGFN1, n (%) |  |  |  | 0.2451 |
| MUT | 9 (31.03) | 6 (42.86) | 3 (20.00) |  |
| WT | 20 (68.97) | 8 (57.14) | 12 (80.00) |  |
| NEB, n (%) |  |  |  | 0.6999 |
| MUT | 9 (31.03) | 5 (35.71) | 4 (26.67) |  |
| WT | 20 (68.97) | 9 (64.29) | 11 (73.33) |  |
| FLG, n (%) |  |  |  | 1 |
| MUT | 8 (27.59) | 4 (28.57) | 4 (26.67) |  |
| WT | 21 (72.41) | 10 (71.43) | 11 (73.33) |  |
| ARID1A, n (%) |  |  |  |  |
| MUT | 7 (24.14) | 6 (42.86) | 1 ( 6.67) | 0.0352 |
| WT | 22 (75.86) | 8 (57.14) | 14 (93.33) |  |

MUT, mutant; PD, progressive disease; PR, partial response; SD, stable disease; WT, wild type.

Figure S1. Patient flow diagram





Figure S2. Flowchart of conversion therapy


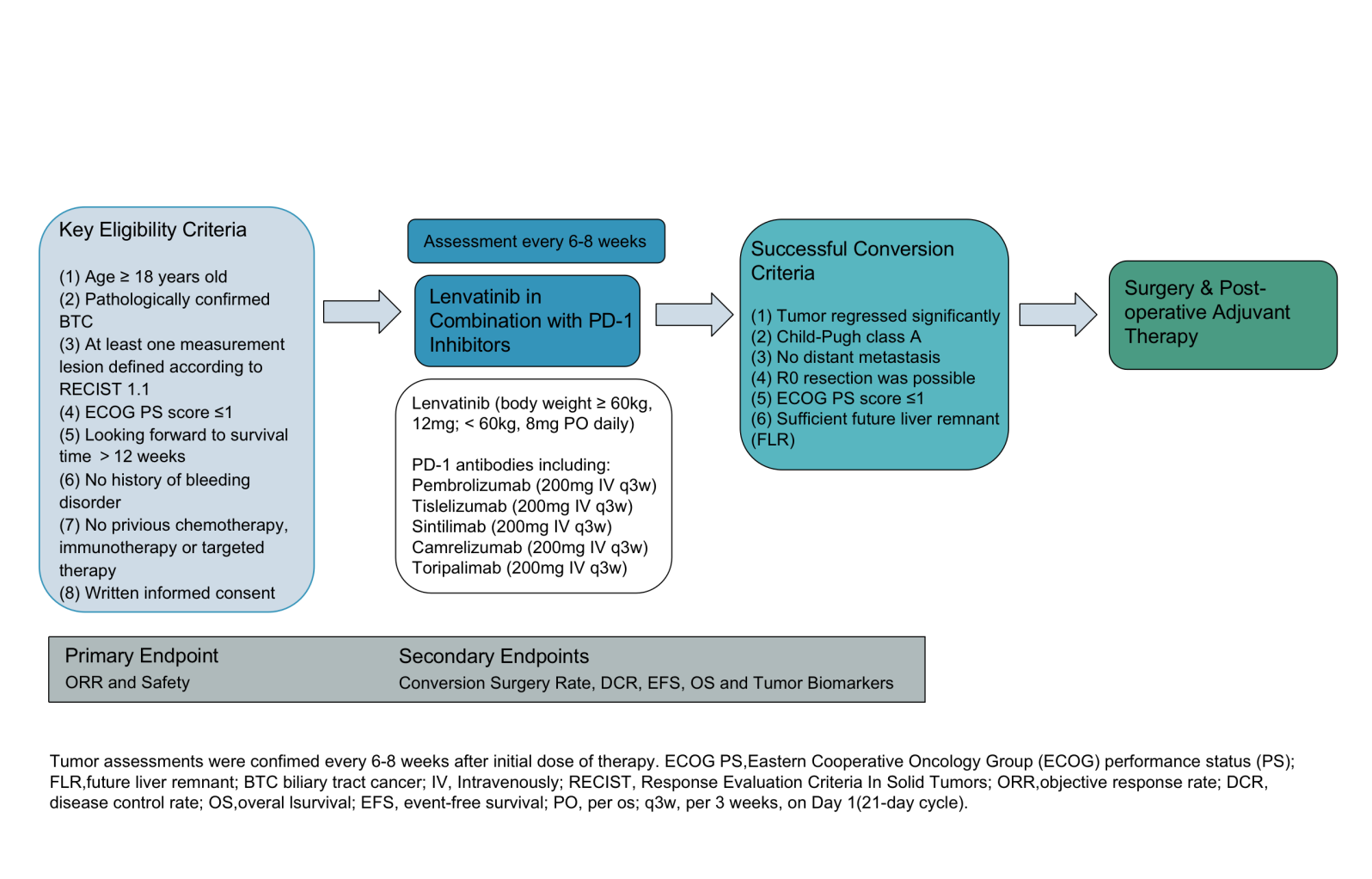


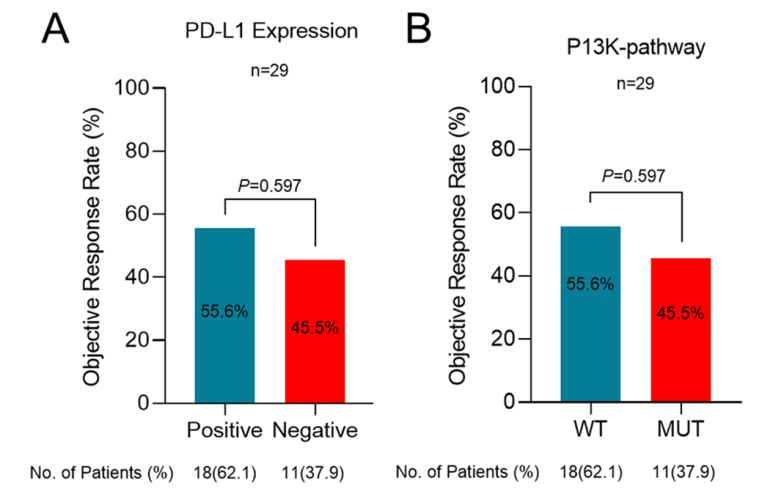


Figure S3. Clinical response in relation to tumor biomarkers in patients with initial unresectable BTC. (A) PD-L1 positive status was defined as the presence of membrane staining of any intensity in 1% or more of tumor cells or immune cells by immunohistochemistry (IHC) staining. (B) Positive P13K-pathway mutation was defined as the abnormality caused by any gene mutation in the pathway.


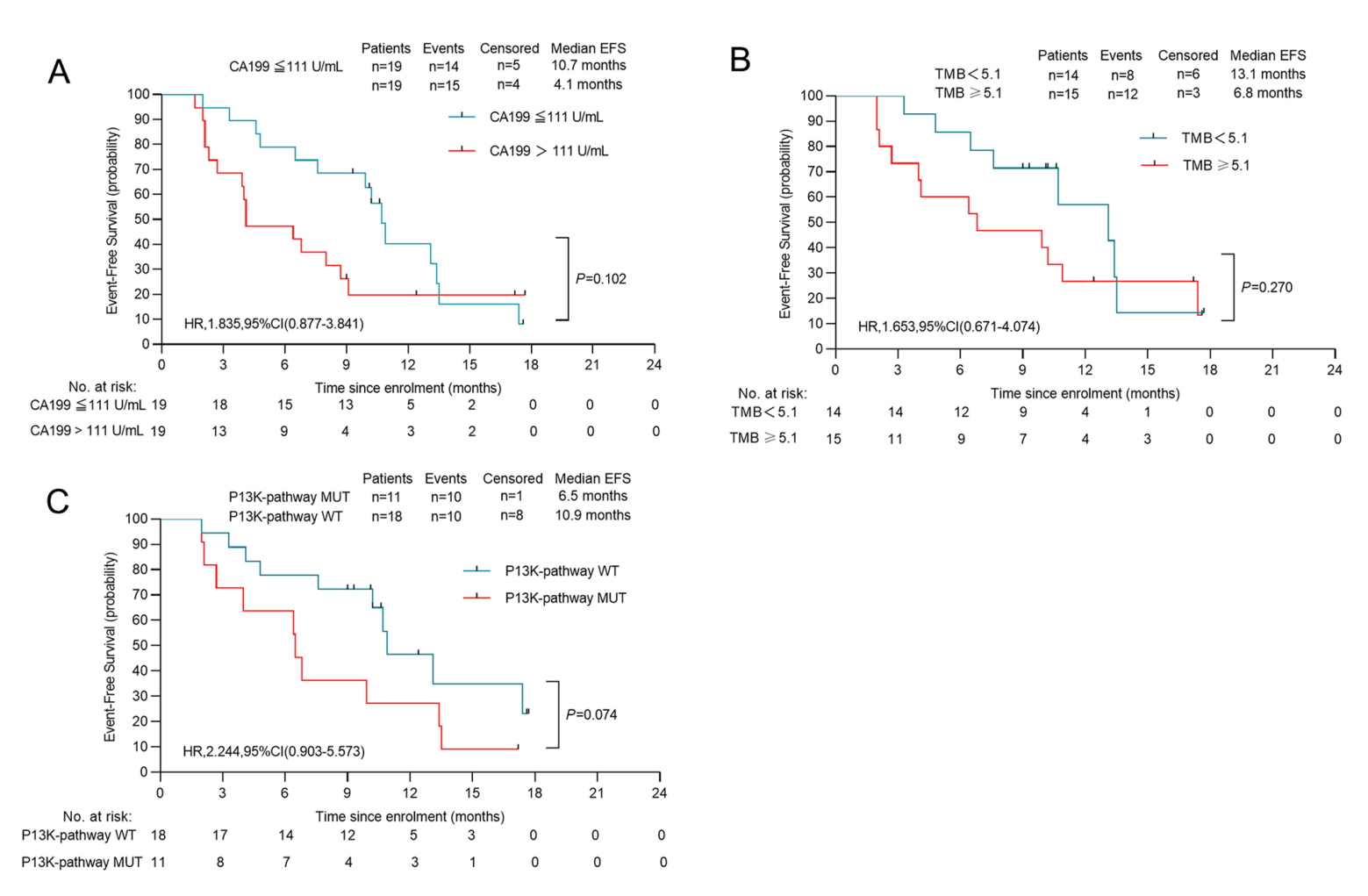


Figure S4. (A) Event-free survival of patients of CA199 ≤ 111 U/ml or CA199＞111 U/ml (n=38). (B) Event-free survival of patients of TMB＜5.1 mutations/Mbp or TMB ≥ 5.1 mutations/Mbp (n=29). (C) Event-free survival of patients of P13K-pathway wide type or P13K-pathway mutation (n=29). Probability of survival is shown at indicated time points. Censored patients are marked with a vertical line in the graph. Numbers of patients at risk at indicated time points are shown below the x-axis.
